# Serum periostin levels in myocardial infarction: Findings from forensic autopsy cases

**DOI:** 10.1371/journal.pone.0356533

**Published:** 2026-08-17

**Authors:** Atsushi Yamada, Kana Unuma, Osamu Kitamura

**Affiliations:** 1 Department of Forensic Medicine, Graduate School of Medical and Dental Sciences, Institute of Science Tokyo (ST), Tokyo, Japan; 2 Department of Legal Medicine, Kyorin University School of Medicine, Tokyo, Japan; Osaka University Graduate School of Medicine, JAPAN

## Abstract

Acute myocardial infarction (AMI) remains one of the leading causes of sudden death with significant implications in both clinical and forensic practice. In forensic settings, AMI diagnosis traditionally relies on gross and histopathological findings. However, when ischemic changes are subtle or insufficiently developed, various cardiac biomarkers have been investigated to support postmortem diagnosis. Periostin, a matricellular protein involved in tissue remodeling and repair, has recently garnered attention for its role in cardiac pathology. Emerging evidence suggests that serum periostin may be a valuable biomarker for assessing cardiac injury and predicting outcomes after AMI. This study aimed to evaluate serum periostin levels in forensic autopsy cases of AMI. The subjects were 80 autopsy cases, categorized into AMI (n = 46), fatal asthma (n = 16), and traumatic deaths (n = 18). Serum periostin levels were significantly elevated in AMI compared with asthma and trauma cases, with median values of 728.50 ng/mL (IQR 465.25–1139.75) in AMI, 410.50 ng/mL (IQR 253.00–756.00) in asthma, and 386.50 ng/mL (IQR 229.75–532.50) in trauma. Serum periostin showed no significant correlation with postmortem interval and was minimally influenced by patients’ characteristics or comorbidities. These findings provide baseline data on serum periostin levels in forensic autopsy cases and suggest that periostin measurement may assist in the postmortem evaluation of AMI.

## 1. Introduction

Acute myocardial infarction (AMI) remains a major cause of sudden death with significant implications in both clinical and forensic practice. In forensic settings, AMI diagnosis primarily relies on gross and histopathological findings. However, when ischemic changes are insufficiently developed, these morphological indicators may be inconclusive, thus presenting a substantial diagnostic challenge [1]. To overcome these limitations, various cardiac biomarkers have been investigated to support postmortem diagnosis. Among them, cardiac troponin T and I (cTnT and cTnI) measured in serum or pericardial fluid have gained acceptance as valuable tools for AMI diagnosis [1–3]. Furthermore, recent studies have demonstrated that combining multiple biomarkers such as cTnI, creatine kinase MB (CK-MB), B-type natriuretic peptide (BNP), lactate dehydrogenase (LDH), and α-hydroxybutyrate dehydrogenase improves diagnostic accuracy [2]. Reliable biomarkers that remain stable in forensic settings and are minimally affected by postmortem changes would therefore be particularly valuable in forensic investigations [4,5]. Periostin, a multifunctional matricellular protein initially identified as osteoblast-specific factor 2, is a secreted extracellular matrix (ECM) protein that plays a critical role in tissue remodeling and repair [6–9]. Increasing evidence suggests that periostin is involved in cardiac pathology, particularly in the structural and functional remodeling of the myocardium after AMI [10–12]. From a diagnostic perspective, serum periostin has recently emerged as a potential biomarker for predicting the prognosis and treatment response after AMI [13–19]. However, its relevance in forensic settings has not been well characterized. Additionally, periostin is implicated in airway remodeling through type 2 helper T (Th2) inflammation and has been proposed as an indicator of disease activity and control in bronchial asthma [20–22]. Considering these properties, this study aimed to evaluate serum periostin levels in forensic autopsy cases and provide baseline data on its potential applicability in postmortem investigations.

## 2. Methods

### 2.1. Subjects

The characteristics of the study subjects are summarized in Table 1. This study included 80 patients who underwent a medicolegal autopsy at our institution between 01/04/2015 and 31/08/2025. Subjects were categorized based on the cause of death as 1) AMI, 2) fatal asthma, and 3) traumatic death due to accident or suicide.

**Table 1:** Characteristics of the study subjects (n = 80).

	AMI	Asthma	Trauma
Number of subjects (women/men)	46 (6/40)	16 (4/12)	18 (5/13)
Age [years]	57.28 ± 17.18	56.31 ± 16.08	58.06 ± 22.51
BMI	25.97 ± 5.75	22.43 ± 4.43	21.25 ± 4.55
Heart weight [g]	467.67 ± 109.45	359.00 ± 68.72	367.22 ± 106.27
PM interval [hours]	30.98 ± 17.75	35.00 ± 20.32	34.50 ± 21.10
Serum periostin levels [ng/mL]	728.50 (IQR, 465.25–1139.75)	410.50 (IQR 253.00–756.00)	386.50 (IQR 229.75–532.50)

Age, BMI, heart weight, and PM interval are presented as the mean ± standard deviation (SD), while serum periostin levels are presented as the median and interquartile range (IQR). AMI, acute myocardial infarction; BMI, body mass index; PM interval, post-mortem interval.

**Fig 1:**
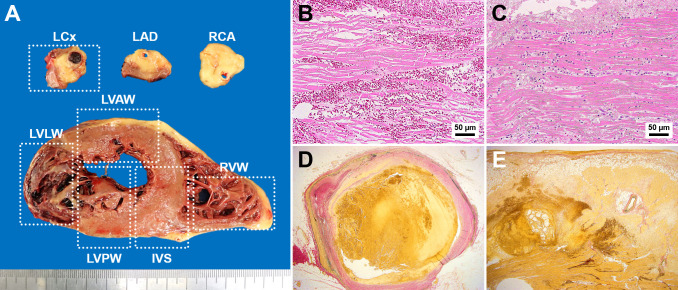
Representative findings of AMI used for diagnostic criteria and sampling. (A) Horizontal cross-section of AMI case showing five myocardial regions (LVAW, LVLW, IVS, RVW) outlined, and LCx with a coronary thrombus identified and sampled. (B) Histological features of early ischemic injury, including wavy myocardial fibers without inflammatory infiltration, interstitial edema, and hemorrhage (H&E stain). (C) Advanced ischemic injury with extensive neutrophilic infiltration (H&E stain). (D) Coronary artery thrombus in LCx (EVG stain). (E) Example of myocardium without fibrotic scarring, evaluated by EVG stain for re-MI assessment. AMI, acute myocardial infarction; LAD, left anterior descending artery; LCx, left circumflex artery; RCA, right coronary artery; LVAW, anterior wall of the left ventricle; LVLW, lateral wall of the left ventricle; LVPW, posterior wall of the left ventricle; IVS, interventricular septum; RVW, right ventricular wall; H&E, Hematoxylin and eosin; EVG, Elastic Van Gieson; re-MI, recurrent myocardial infarction.

We selected cases of AMI in which both the culprit artery and myocardial histopathological changes were identified. The presence of a coronary thrombus and thin-cap fibroatheroma in the left anterior descending artery (LAD), left circumflex artery (LCx), or right coronary artery (RCA) was confirmed. Three to four horizontal cross-sections from the apex to the mid-ventricle were macroscopically examined. Short-axis sections of the coronary arteries were also evaluated. To identify the primary infarct region, histopathological examination was performed by dividing the myocardium into five distinct regions (Fig 1A): anterior wall of the left ventricle (LVAW), lateral wall of the left ventricle (LVLW), posterior wall of the left ventricle (LVPW), interventricular septum (IVS), and right ventricular wall (RVW). Each region was assessed individually to determine the presence and extent of early ischemic changes. Histopathological evidence of ischemic changes was supported by findings, including coagulation necrosis characterized by loss of cross-striations, contraction band formation, interstitial edema, hemorrhage, and early neutrophilic infiltration; progressive coagulation necrosis with nuclear pyknosis and marginal contraction bands; and ultimately, complete loss of nuclei and cross-striations accompanied by extensive neutrophilic infiltration (Fig 1B, C). If a thrombus was identified in the coronary artery, it was subjected to histopathological examination, primarily to exclude vasculitis involvement (Fig 1D). Furthermore, cases with fibrotic replacement of myocardial tissue attributable to recurrent myocardial infarction (re-MI) were assessed and classified according to the presence or absence of fibrotic scarring (Fig 1E). To analyze the relationship between serum periostin levels and the anatomical characteristics of MI, we compared periostin concentrations according to both the culprit coronary artery and the primary infarct region among AMI cases (n = 46). Cases presumed to involve fatal arrhythmia, characterized by only coronary artery stenosis due to stable plaques without morphological abnormalities of the myocardium, were excluded from this study.

Asthma was diagnosed based on specific findings and pre-mortem symptoms [23,24]. Traumatic deaths were used as the reference group because they were considered unlikely to involve systemic inflammatory or fibrotic conditions that may influence serum periostin levels. Traumatic deaths included falls from height, falling down, traffic accidents, blunt assault-related homicides, and self-inflicted stabbings. In both the asthma and trauma groups, individuals with evidence of coronary atherosclerosis or MI were excluded. Furthermore, all study subjects underwent comprehensive macroscopic and histopathological examinations of multiple organs, including the brain, heart, lungs, liver, kidneys, spleen, pancreas, pituitary gland, thyroid gland, thymus, adrenal glands, gastrointestinal tract, aorta, and reproductive organs. Cases were screened to exclude those with evidence of atopic dermatitis, neoplasm, aneurysmal disease, and diseases primarily characterized by fibrosis, such as pulmonary fibrosis and systemic sclerosis. Two forensic pathologists comprehensively evaluated all cases, including one author with subspecialty expertise in dermatology.

The number of subjects (women/men) was as follows: AMI, 46 (6/40); asthma, 16 (4/12); and trauma, 18 (5/13). The mean ± standard deviation (SD) ages of the subjects were as follows: AMI, 57.28 ± 17.18 years; asthma, 56.31 ± 16.08 years; and trauma, 58.06 ± 22.51 years. The mean ± SD body mass index (BMI) of the subjects was as follows: AMI, 25.97 ± 5.75; asthma, 22.43 ± 4.43; and trauma, 21.25 ± 4.55. The mean ± SD heart weight of the subjects was as follows: AMI, 467.67 ± 109.45 g; asthma, 359.00 ± 68.72 g; and trauma, 367.22 ± 106.27 g. The mean ± SD PM intervals of the subjects were as follows: AMI, 30.98 ± 17.75 h; asthma, 35.00 ± 20.32 h; and trauma, 34.50 ± 21.10 h.

Written informed consent to use individual data for academic publication was obtained from the bereaved family, ensuring the utmost respect for privacy and confidentiality. All data were fully anonymized prior to analysis. The study was approved by the ethics committee of Kyorin University School of Medicine (registration number: R07-2557). Data were accessed for research purposes between 21/04/2025 and 31/08/2025.

### 2.2. Serum periostin levels

A blood sample was obtained from the right atrial appendage, and blood cells were separated immediately by centrifugation. The supernatants were collected and stored at −80 °C until further analysis. Samples showing marked hemolysis were excluded from analysis. No repeated freeze–thaw cycles were performed before measurement. Serum periostin levels were measured by outsourced providers using an enzyme-linked immunosorbent assay (SRL, Inc., Tokyo, Japan).

### 2.3. Comparison of data dispersion among groups

**Fig 2:**
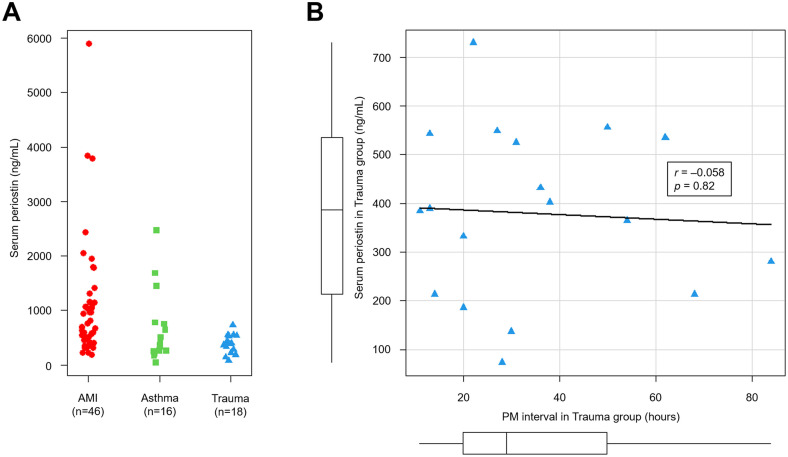
(A) Scatter plot illustrating the serum periostin levels in subjects with AMI, asthma, and trauma (n = 80). Based on this comparison, the trauma group serves as a reference for the AMI and asthma groups. (B) In the trauma groups, correlation analysis revealed no significant association between serum periostin levels and PM interval (r = −0.058, p = 0.82). AMI, acute myocardial infarction. PM interval, post-mortem interval.

Serum periostin levels in the AMI, asthma, and trauma groups were compared, and scatter plots were used to visualize the data dispersion (Fig 2A). Based on this comparison, we evaluated the suitability of the trauma group as a reference for the AMI and asthma groups.

### 2.4. Validation of the effects of postmortem changes in the serum periostin level

To ensure the integrity of serum periostin measurements, we evaluated the effects of postmortem degradation on the samples. In the control group, the correlations between serum periostin levels and PM intervals were evaluated.

### 2.5. Validation of the differences in serum periostin levels based on physical characteristics and comorbidities in AMI

Correlations between serum periostin levels and physical characteristics, including age, BMI, and heart weight (HW), were evaluated. Clinical parameters were categorized according to major cardiovascular risk factors as follows: diabetes mellitus history (present: n = 11, absent: n = 35), dyslipidemia history (present: n = 11, absent: n = 35), and hypertension history (present: n = 18, absent: n = 28).

### 2.6. Statistical analysis

Unless otherwise indicated, the data are presented as the median and interquartile range (IQR). To meet the assumptions of parametric statistical tests, skewed continuous variables, such as serum periostin levels, were logarithmically transformed before analysis. Prior to statistical analysis, normality of the data distribution was assessed using the Shapiro–Wilk test. A t-test was applied to compare normally distributed continuous variables between two groups. One-way analysis of variance (ANOVA) with Bonferroni adjustment was utilized to compare normally distributed continuous variables between more than three groups. Receiver operating characteristic (ROC) curve analysis was conducted to assess the diagnostic utility of serum periostin levels. Correlations were evaluated using Pearson’s product–moment correlation coefficient. Receiver operating characteristic (ROC) curve analysis was conducted to assess the diagnostic utility of serum periostin levels. All statistical analyses were conducted with EZR, a graphical user interface for R (The R Foundation for Statistical Computing, Vienna, Austria) modified for biostatistics [25]. The results were significant at *p < 0.05, **p < 0.01, and ***p < 0.001.

## 3. Results

### 3.1. Serum periostin levels in subjects

The serum periostin levels in the subjects were as follows: AMI, 728.50 ng/mL (IQR, 465.25–1139.75); asthma, 410.50 ng/mL (IQR 253.00–756.00); and trauma, 386.50 ng/mL (IQR 229.75–532.50) (Table 1).

### 3.2. Postmortem changes in serum periostin levels

Correlation analysis of the trauma groups (mean ± SD PM interval: 34.50 ± 21.10 h; range: 11–84 h) revealed no significant correlation between serum periostin levels and PM intervals (r = −0.058, p = 0.82; Fig 2B).

### 3.3. Comparison of serum periostin levels among groups

**Fig 3:**
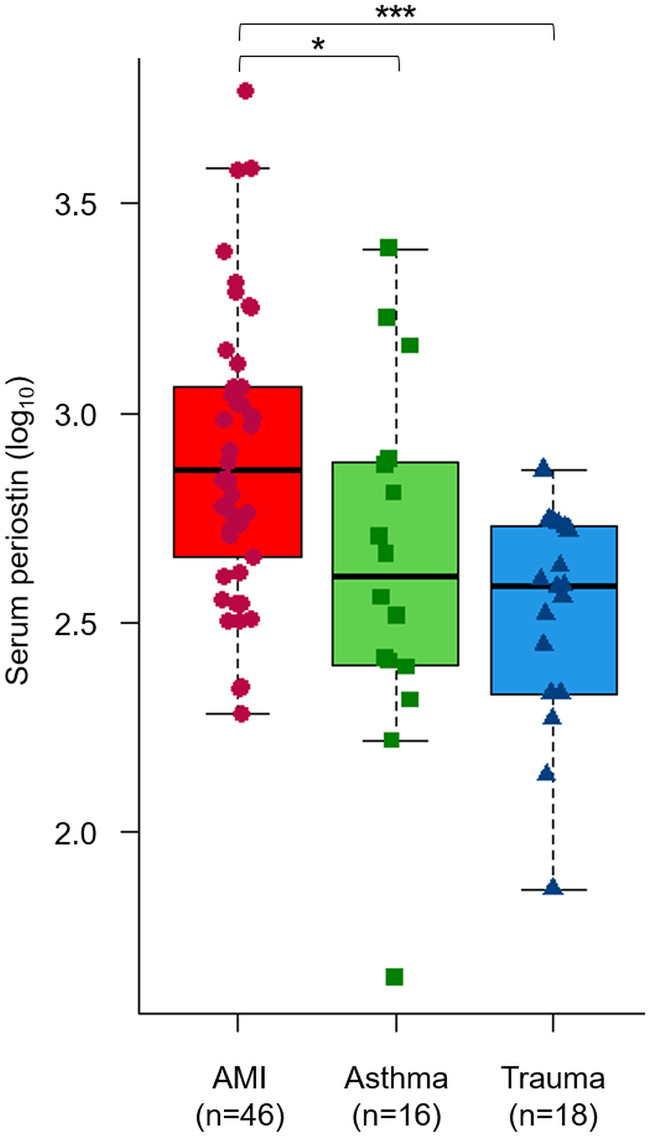
Comparison of serum periostin levels among AMI, asthma, and trauma groups. One-way analysis of variance (ANOVA) with Bonferroni adjustment revealed highly significant contrasts between AMI and asthma (p = 0.03015) and between AMI and trauma (p = 0.00048) groups. No significant difference was observed between asthma and trauma groups (p = 0.98659). AMI, acute myocardial infarction.

Serum periostin levels in the AMI group were significantly higher than those in the asthma and trauma groups (Fig 3). One-way ANOVA with Bonferroni adjustment revealed highly significant contrasts between AMI and asthma (p = 0.03015) and between AMI and trauma (p = 0.00048). Asthma and trauma showed no significant differences (p = 0.98659).

### 3.4. Correlations among serum periostin levels, categorical variables, and comorbidities related to AMI

**Fig 4:**
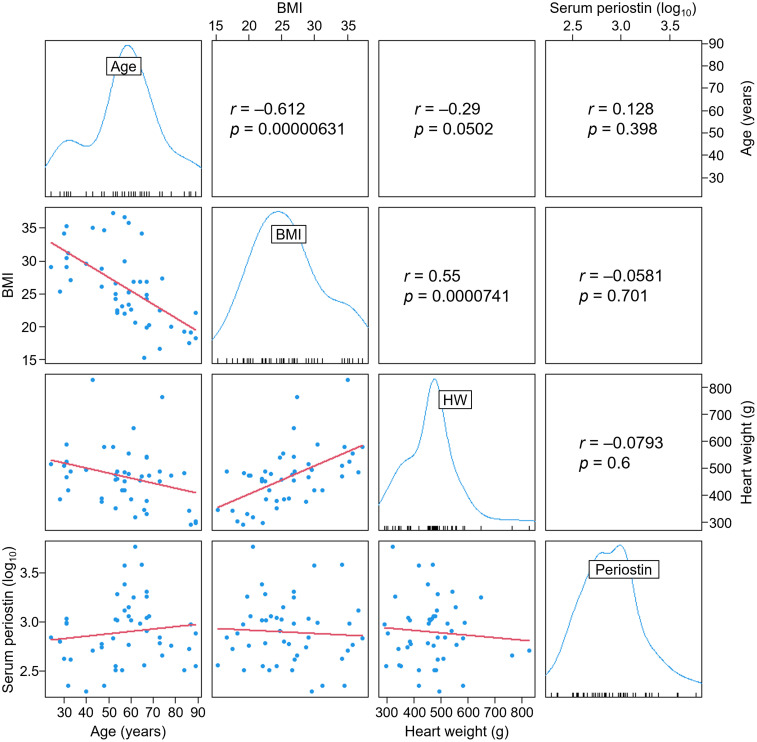
Correlation analysis between serum periostin levels and physical parameters, including age (r = 0.128, p = 0.398), BMI (r = –0.0581, p = 0.701), and HW (r = –0.0793, p = 0.6). BMI, body mass index; HW, heart weight.

Correlation analysis revealed no significant association between serum periostin levels and physical parameters, including age (r = 0.128, p = 0.398), BMI (r = –0.0581, p = 0.701), and HW (r = –0.0793, p = 0.6; Fig 4). These findings suggest that general physiological characteristics do not substantially influence serum periostin levels. Patients with AMI were grouped according to clinical parameters, and serum periostin levels in each group were compared (Table 2). The values for women (n = 6) and men (n = 40) were 759.5 ng/mL (IQR, 553.25–1220) and 728.5 ng/mL (IQR, 442.75–1119.25), respectively, with no significant differences between the two groups (p = 0.497). In cases with a history of diabetes mellitus (n = 11), the values were 676 ng/mL (IQR, 489–1101), compared with 760 ng/mL (IQR, 463–1129.5) in those without diabetes mellitus (n = 35; p = 0.57). For dyslipidemia, the values were 573 ng/mL (IQR, 434.5–1055.5) in cases with a positive history (n = 11) and 813 ng/mL (IQR, 526–1233.5) in those without dyslipidemia (n = 35; p = 0.165). Similarly, subjects with a history of hypertension (n = 18) were 890 ng/mL (IQR, 512.75–1152.25), compared with 694.5 ng/mL (IQR, 415.75–1083.5) in those without hypertension (n = 28; p = 0.89). Based on the presence or absence of major cardiovascular risk factors, no statistically significant differences in serum periostin levels were observed.

**Table 2:** Association between serum periostin levels and clinical parameters in AMI (n = 46).

Variables	Serum periostin levels [ng/mL]				*p*-value
Sex	Women (n = 6)		Men (n = 40)		0.497
	759.5 (IQR, 553.25–1220)		728.5 (IQR, 442.75–1119.25)		
Diabetes mellitus	Present (n = 11)		Absent (n = 35)		0.57
	676 (IQR, 489–1101)		760 (IQR, 463–1129.5)		
Dyslipidemia	Present (n = 11)		Absent (n = 35)		0.165
	573 (IQR, 434.5–1055.5)		813 (IQR, 526–1233.5)		
Hypertension	Present (n = 18)		Absent (n = 28)		0.89
	890 (IQR, 512.75–1152.25)		694.5 (IQR, 415.75–1083.5)		
Culprit artery	LAD (n = 32)	LCx (n = 6)	RCA (n = 8)		0.97–1.00
	694.5 (IQR, 415.75–1191)	554 (IQR, 397–891)	1047 (IQR, 768.25–1087.75)		
Primary infarct region	LVAW (n = 32)	LVLW (n = 6)	LVPW (n = 3)	IVS (n = 5)	1.00
	694.5 (IQR, 415.75–1191)	554 (IQR, 397–891)	1045 (IQR, 786–1055.5)	1049 (IQR, 813–1153)	
Fibrosis following re-MI	Present (n = 13)		Absent (n = 33)		0.447
	697 (IQR, 451–1045)		760 (IQR, 508–1150)		

Data are presented as the median and interquartile range (IQR). AMI, acute myocardial infarction; LAD, left anterior descending artery; LCx, left circumflex artery; RCA, right coronary artery; LVAW, anterior wall of the left ventricle; LVLW, lateral wall of the left ventricle; LVPW, posterior wall of the left ventricle; IVS, interventricular septum; RVW, right ventricular wall; re-MI, recurrent myocardial infarction. A *t*-test was applied to normally distributed continuous variables. One-way ANOVA was utilized to compare normally distributed continuous variables between more than three groups.

### 3.5. Correlations among serum periostin levels, culprit artery, infarct region, and re-MI

No statistically significant difference was observed in serum periostin levels among groups with different culprit arteries. The median serum periostin levels in the subjects were as follows: LAD (n = 32), 694.5 ng/mL (IQR, 415.75–1191); LCx (n = 6), 554 ng/mL (IQR, 397–891); and RCA (n = 8), 1047 ng/mL (IQR, 768.25–1087.75) (p = 0.97–1.00). Similarly, no significant differences were observed in serum periostin levels among primary infarct regions. The serum periostin levels in the subjects were as follows: LVAW (n = 32), 694.5 ng/mL (IQR, 415.75–1191); LVLW (n = 6), 554 ng/mL (IQR, 397–891); LVPW (n = 3), 1045 ng/mL (IQR, 786–1055.5); and IVS (n = 5), 1049 ng/mL (IQR, 813–1153) (p = 1.00). Based on the presence or absence of myocardial fibrotic scarring, no significant differences were identified between the two groups (Table 2). The median serum periostin levels in the subjects were as follows: 697 ng/mL (IQR, 451–1045) in the group with fibrotic scarring (n = 13) and 760 ng/mL (IQR, 508–1150) in the group without fibrotic scarring (n = 33, p = 0.447).

### 3.6. Diagnostic utility of periostin levels in AMI

**Fig 5:**
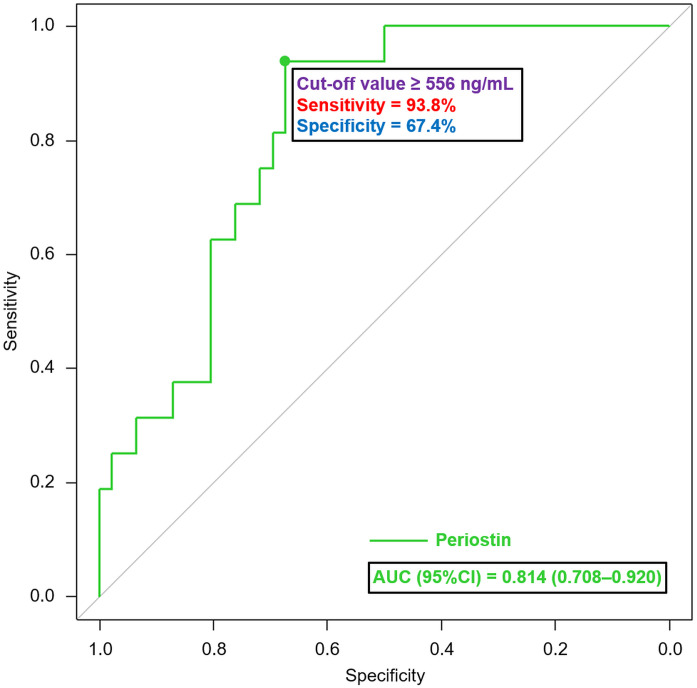
ROC curve analysis of serum periostin measurements for the diagnosis of AMI with a cut-off value of 556 ng/mL. The sensitivity was 93.8%, and the specificity was 67.4%, with an AUC of 0.814 (95% CI: 0.708–0.920). ROC, receiver operating characteristic; AUC, area under the curve; CI, confidence interval.

ROC curve analysis was performed using a cut-off value of 556 ng/mL (Fig 5). Serum periostin measurements demonstrated a sensitivity of 93.8%, specificity of 67.4%, and area under the curve (AUC) of 0.814 (95% confidence interval, CI, 0.708–0.920). When measuring serum periostin levels with a cut-off value of 556 ng/mL, the diagnostic indices were as follows: positive predictive value (PPV), 0.939; negative predictive value (NPV), 0.516; diagnostic accuracy, 0.734; positive likelihood ratio (LR+), 6.065; and negative likelihood ratio (LR-), 0.367.

## 4. Discussion

### 4.1. Postmortem impact on serum periostin levels

In forensic settings, the interpretation of biochemical markers is often affected by postmortem degradation, which compromises sample integrity and limits the applicability of clinical reference ranges [4,5]. To address this issue, we assessed the impact of the PM interval on serum periostin levels. In this study, no significant correlation was observed between serum periostin levels and PM intervals in the trauma group, which was set as the control group (Fig 2B). However, a gradual downward trend in serum periostin levels was observed with increasing PM interval, suggesting that postmortem degradation may influence periostin measurements. Therefore, although serum periostin may remain relatively stable for up to 84 h, these findings should be interpreted cautiously in cases with advanced postmortem degradation.

### 4.2. Correlation among serum periostin levels, categorical variables, and comorbidities related to AMI

In this study, serum periostin levels demonstrated minimal susceptibility to intrinsic biological variations. The absence of significant correlations between serum periostin levels and categorical variables, including sex, age, BMI, HW, and cardiovascular risk factors (diabetes mellitus, dyslipidemia, and hypertension), suggests that baseline patient characteristics do not substantially influence serum periostin. These findings suggest that serum periostin may provide useful supportive information compared with conventional biomarkers that are susceptible to demographic factors [26–28], particularly in forensic settings where clinical background information is often limited or unavailable.

### 4.3. Correlation among serum periostin levels, infarct region, and re-MI

Previous experimental studies have demonstrated that periostin is significantly upregulated in the infarct region after AMI [29], where it contributes to fibroblast activation, collagen synthesis, and ECM stabilization, all of which are crucial for myocardial repair and formation of fibrotic scar tissue [30–33]. In animal models, exogenous periostin administration has improved cardiac function following AMI [31,32]. However, contrasting studies suggest that periostin may contribute to maladaptive remodeling and deterioration of cardiac function [18,34–36]. This duality highlights the complex role of periostin, which may contribute to both tissue repair and subsequent fibrosis, ultimately leading to maladaptive remodeling [37]. Considering these backgrounds, we hypothesized that serum periostin levels might increase with increasing infarct size or region of myocardial injury or be influenced by prior infarction and fibrotic remodeling. However, in the current study, no significant correlation was observed between serum periostin levels and anatomical characteristics of MI, which did not support our hypothesis (Table 2). The lack of association between serum periostin levels and either the infarct region or fibrotic scarring suggests that postmortem periostin evaluation reflects the presence of AMI, rather than its severity or chronicity. Future studies using more homogeneous cohorts, such as cases limited to LAD-related AMI, together with detailed histopathological stratification according to the temporal phase of AMI, may help clarify whether serum periostin levels reflect infarct extent, severity, or the progression of myocardial remodeling after AMI.

### 4.4. Diagnostic utility of periostin

A ROC curve analysis with a cut-off value of 556 ng/mL for serum periostin measurements demonstrated a sensitivity of 93.8% and specificity of 67.4%, with an AUC of 0.814 (Fig 5). The PPV and NPV were 0.939 and 0.516, respectively. The overall diagnostic accuracy was 0.734, with an LR+ of 6.065 and an LR- of 0.367. These findings suggest that serum periostin may have potential utility in the postmortem evaluation of AMI. The relatively high LR+ value indicates that elevated periostin levels are associated with an increased likelihood of AMI. However, the lower NPV and modest LR− values suggest that serum periostin levels alone may not be sufficient to reliably exclude AMI when values are below the cut-off threshold. Taken together, these findings indicate that serum periostin measurement may provide supportive diagnostic information for AMI, particularly when interpreted alongside autopsy findings and conventional biomarkers.

### 4.5. Interpretation of elevated periostin levels in the context of comorbidities

Several non-cardiac conditions are known to elevate serum periostin levels independently. Periostin is a multifunctional matricellular protein that contributes to ECM formation and modulates inflammation through downstream pathways associated with Th2 immune responses [38]. Elevated serum periostin levels have been reported in various fibrotic and Th2-mediated inflammatory diseases, including idiopathic pulmonary fibrosis [39], systemic sclerosis [40], aortic remodeling [9], atopic dermatitis [41], and bronchial asthma [20–22]. Furthermore, periostin plays a key role in the development of cancer-associated fibrosis and tumor microenvironment, with emerging evidence supporting its potential in cancer surveillance and treatment response, particularly in malignancies such as non-small cell lung carcinoma and breast cancer [42–44]. Considering these broad pathological associations, elevated serum periostin levels should be interpreted with caution, particularly in individuals with comorbid fibrotic conditions, Th2-mediated inflammation, or neoplastic diseases.

### 4.6. Potential of periostin as a biomarker of fatal asthma

In this study, difference in serum periostin levels between the asthma and trauma groups did not reach statistical significance (Fig 3). These findings are inconsistent with those of previous studies, which suggest elevated serum periostin levels in patients with bronchial asthma [20–22]. However, the dot plot for the asthma group revealed a wide distribution of serum periostin levels, ranging from extremely high to values equivalent to those obtained for the trauma group (Fig 2A). Recent research has progressively highlighted the heterogeneity of asthma, emphasizing that understanding the phenotype can predict the efficacy of treatment and prognosis [45]. Particularly, Th2-low asthma is a phenotype refractory to conventional therapies and is implicated in asthma-related sudden death [46]. Given the limited role of periostin in Th2-low–mediated inflammation, the phenotypic heterogeneity observed among fatal asthma cases may reflect discrepancies with prior studies of living individuals.

### 4.7. Study limitations

This study has several limitations. First, only cases with histopathological evidence of AMI were included owing to the study design. Given the known kinetics of periostin expression, the duration from AMI onset to death in this study was estimated to be approximately 1–3 days. Animal studies have demonstrated periostin upregulation approximately four days post-AMI; human data suggest that serum periostin levels increase as early as 1–3 days post-AMI [14,16,47], which aligns with our inclusion criteria. Therefore, serum periostin levels observed in present study may primarily reflect subacute myocardial remodeling and repair process rather than hyperacute ischemic injury itself. Future studies should investigate cases of sudden death shortly after onset, with confirmed coronary occlusion but without morphological myocardial changes, to assess the diagnostic value of periostin in the earliest phase of AMI. Secondly, the subject data relied on police investigations, which may lack comprehensive details on potential confounders, including comorbidities, smoking, medication use, and a history of cardiopulmonary resuscitation. Thirdly, although serum periostin levels did not show a statistically significant correlation with PM interval, a gradual downward trend was observed (Fig 2B), suggesting that postmortem degradation may influence serum periostin measurements in some cases. Therefore, the present findings should be interpreted cautiously, in individuals with advanced postmortem degradation. Fourthly, direct comparison with established cardiac biomarkers, such as cTnT, cTnI, and CK-MB is not evaluated. Therefore, the incremental diagnostic value of serum periostin beyond conventional biomarkers could not be determined in the present study. Future studies incorporating simultaneous measurement of periostin and established cardiac biomarkers in larger forensic cohorts will be necessary to clarify the potential complementary role of periostin in the postmortem evaluation of AMI. Finally, the relatively small sample size and the difficulty in obtaining truly healthy postmortem controls remain important limitations of this study. Future multicenter collaborative studies with larger cohorts and more standardized control populations will be necessary to further validate the forensic applicability of serum periostin measurements.

## 5. Conclusion

The present study provides baseline data on serum periostin levels in forensic autopsy cases. Our findings suggest that serum periostin measurement may provide useful supportive information for the postmortem evaluation of AMI in forensic practice. Serum periostin levels may remain relatively stable for up to 84 h postmortem and were not significantly influenced by patient characteristics or major comorbidities. Although periostin is not specific to AMI and may also be elevated in individuals with fibrotic conditions, Th2-mediated inflammatory diseases, aortic remodeling, or neoplastic disorders. The present findings indicate that serum periostin measurement may assist in the postmortem evaluation of AMI when interpreted alongside autopsy findings and other conventional biomarkers.
